# Cerebral malaria: insights from host-parasite protein-protein interactions

**DOI:** 10.1186/1475-2875-9-155

**Published:** 2010-06-09

**Authors:** Aditya Rao, Mayil K Kumar, Thomas Joseph, Gopalakrishnan Bulusu

**Affiliations:** 1 Life Sciences Division, TCS Innovation Labs Hyderabad, Tata Consultancy Services Ltd, 1, Software Units Layout, Madhapur, Hyderabad - 500081, India

## Abstract

**Background:**

Cerebral malaria is a form of human malaria wherein *Plasmodium falciparum*-infected red blood cells adhere to the blood capillaries in the brain, potentially leading to coma and death. Interactions between parasite and host proteins are important in understanding the pathogenesis of this deadly form of malaria. It is, therefore, necessary to study available protein-protein interactions to identify lesser known interactions that could throw light on key events of cerebral malaria.

**Methods:**

Sequestration, haemostasis dysfunction, systemic inflammation and neuronal damage are key processes of cerebral malaria. Key events were identified from literature as being crucial to these processes. An integrated interactome was created using available experimental and predicted datasets as well as from literature. Interactions from this interactome were filtered based on Gene Ontology and tissue-specific annotations, and further analysed for relevance to the key events.

**Results:**

PfEMP1 presentation, platelet activation and astrocyte dysfunction were identified as the key events influencing the disease. 48896 host-parasite along with other host-parasite, host-host and parasite-parasite protein-protein interactions obtained from a disease-specific corpus were combined to form an integrated interactome. Filtering of the interactome resulted in five host-parasite PPI, six parasite-parasite and two host-host PPI. The analysis of these interactions revealed the potential significance of apolipoproteins and temperature/Hsp expression on efficient PfEMP1 presentation; role of MSP-1 in platelet activation; effect of parasite proteins in TGF-β regulation and the role of albumin in astrocyte dysfunction.

**Conclusions:**

This work links key host-parasite, parasite-parasite and host-host protein-protein interactions to key processes of cerebral malaria and generates hypotheses for disease pathogenesis based on a filtered interaction dataset. These hypotheses provide novel and significant insights to cerebral malaria.

## Background

Malaria remains a scourge in the developing world, with the number of fatalities due to the disease estimated at one million every year [1]. *Plasmodium falciparum *is the most fatal of the four Plasmodium species that cause human malaria, accounting for a large proportion of these deaths [2,3]. Cerebral malaria (CM) is a severe form of *P. falciparum *infection, characterized by cerebral complications, such as neuronal damage and coma [3].

Processes such as sequestration, systemic inflammation, haemostasis dysfunction and neuronal damage characterize CM [4,5]. Host-parasite protein interactions are crucial to understanding these processes. For instance, interactions between the parasite protein PfEMP1 and human proteins such as CD36 and inter-cellular adhesion molecule (ICAM-1) expressed in endothelial cells (EC) are critical for sequestration [6]. Sequestration is the adhesion of *P. falciparum*-infected red blood cells (pRBCs) to the EC. Such interactions are known to trigger intracellular signaling cascades within the EC. These affect the expression of key proteins in the blood-brain barrier (BBB) intercellular tight junctions, including zona occludens-1, vinculin and occludin, leading to eventual BBB disruption [6,7].

Protein-protein interactions (PPI) between host and parasite proteins are thus crucial to studying the disease. However, current understanding of the molecular processes involving the host-parasite PPI is limited and the significance of a large number of host-parasite PPI yet to be determined. This work integrates PPI from a multitude of sources to create an integrated PPI landscape, and links some of these interactions to key processes and events of CM. The landscape also includes upstream PPI involving only parasite proteins as well as downstream PPI involving only host proteins that are necessary to understand the triggers and outcomes of these processes and events, respectively.

## Methods

### PPI from predicted datasets

**Figure 1:**
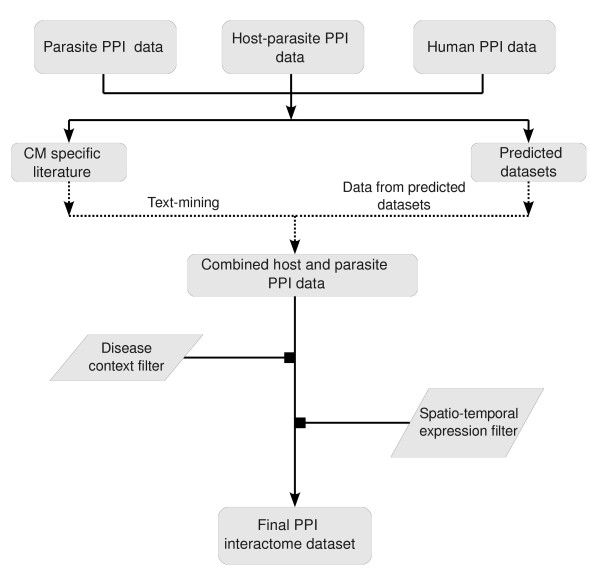
**Workflow followed to obtain the final PPI interactome**.

A number of recent studies predict host-*P. falciparum *PPI [8-11]. PPI datasets were obtained from these studies and a unified set of host-parasite PPI was created. Since each dataset uses a different nomenclature system for the human and parasite proteins, all datasets were transformed to enable comparison and integration using common gene names from sources such as UniProt [12], Ensembl [13] and PlasmoDB [14]. For example, Davis *et al *[8] use the Ensembl protein ID, Dyer *et al *[9] the UniProt entry name, Krishnadev and Srinivasan [10] the NCBI gi code, and Vignali *et al *[11] the UniProt gene names for the human proteins. After this transformation, the various datasets were compared for overlap, and a unified host-*P. falciparum *PPI interactome was created. Figure 1 depicts the schematic work flow followed to create/filter the relevant PPI used in the study.

### CM-specific literature corpus

An automated literature retrieval module was developed using Entrez Programming Utilities [15] to retrieve the list of full-text articles relevant to *P. falciparum*. This article set was further pruned using the MeSH controlled vocabulary to obtain only articles relevant to CM. The resultant set was augmented by articles retrieved from the Google Scholar database using appropriate CM-specific query terms.

Crucial review articles from the literature corpus were used to identify events relevant to the main processes of CM. Furthermore, host-parasite, host-host and parasite-parasite PPI reported in literature were also obtained by analysing this corpus. This was done by first checking for article-level co-occurrence of protein pairs using a utility script implemented in Perl. The script automatically downloads the full-text articles from the respective journal websites as Portable Document Format (PDF) files and converts these to text format using the XPDF conversion utility [16]. All parasite and host proteins that occur in the full-text of each article were identified using dictionary lookup, with PlasmoDB and UniProt/Ensembl being used to create the *P. falciparum *and human protein dictionaries respectively. Only those articles that had at least one protein pair (host-parasite, host-host or parasite-parasite) were considered for further analysis. The PPI obtained from predicted datasets and CM-specific literature corpus were combined to form the integrated interactome consisting of host-parasite, host-host and parasite-parasite PPI.

### Pruning the interactome

Gene Ontology (GO) annotations for process, function and cellular component can be used to filter out false positives from predicted PPI datasets [17]. Using this approach, GO cellular component annotations from PlasmoDB were used to prune the unified PPI interactome. Interactions involving parasite proteins annotated to be present on the pRBC/merozoite surface or reported to be released during schizont rupture [18-20] were only considered. For the human protein annotations, tissue-specific annotations from UniProt were used to prune the interactome. The resultant interactions were further analysed and filtered based on their relevance to the key events that influence the processes of CM, as identified from the key review articles.

## Results

### PPI from predicted datasets

A comparison of the interactions from the predicted PPI datasets demonstrated very little overlap between the various computationally predicted PPI datasets. For example, there were no common interactions between the Vignali and Krishnadev datasets while the Krishnadev and Dyer datasets had only 10 common interactions. Three common interactions between the Dyer and Lee datasets and four common interactions between Krishnadev and Lee datasets were present. A total of 48,896 host- *P. falciparum *PPI were obtained by unifying all the datasets.

### CM-specific corpus analysis

Three events were identified as crucial in influencing sequestration, haemostasis dysfunction, systemic inflammation and neuronal damage, the key processes of CM. They were: PfEMP1 presentation, platelet activation and astrocyte dysfunction. Host-*P. falciparum*, host-host and parasite-parasite PPI reported in literature were obtained by analysing this CM-specific literature corpus. 48,896 host-parasite PPI from the various PPI datasets along with the host-parasite, host-host and parasite-parasite PPI obtained from the literature analysis were combined to form an integrated interactome.

After pruning, the final PPI interactome consisted of five host-parasite PPI, six parasite-parasite and two host-host PPI. The host-parasite PPI are:

• Parasite protein ETRAMP5 (early transcribed membrane protein 5; PFE1590w) with the human apolipoproteins apoA1, apoB and apoE

• Human glycoprotein integrin gpIIIa with the parasite merozoite surface protein MSP-1 (PFI1475w)

• Interactions between host TGF-β~TGF-β receptors and certain parasite proteins such as PF11_0188, PFC0755 etc

The parasite-parasite PPI are:

• Parasite proteins ETRAMP5 (PFE1590w) and PfHsp40 (PF14_0700). Also, PfHsp40 has a direct interaction with PfHsp70 (PF08_0054) and an indirect interaction with PfHsp86 (PF07_0029).

The host-host PPI is:

• An interaction between the human serum albumin (HSA) and TGF-β receptors

### Analysis

#### *PfEMP1 *presentation

The *P. falciparum *protein ETRAMP5 is seen to interact with the human apolipoproteins apoA1, apoB and apoE. ETRAMP5 is responsible for the junction formation between the tubulovesicular network (TVN) and the pRBC [20,21]. ETRAMP5 is known to be critical for efficient PfEMP1 presentation on the pRBC membrane [20,22]. It is known that both high and low density serum lipoproteins play a crucial role in efficient PfEMP1 presentation by mediating lipid transport and thereby assisting PfEMP1 transport from the Maurer's clefts to the pRBC surface [22,23]. This lipoprotein mediated lipid transport occurs via specific apolipoproteins [24], and it has been speculated that parasite proteins might influence this transport via lipoprotein binding [25]. It is thus possible that interactions between ETRAMP5 and the human apolipoproteins apoA1, apoB and apoE might play a crucial role in lipid transport, thereby influencing efficient PfEMP1 presentation.

**Figure 2:**
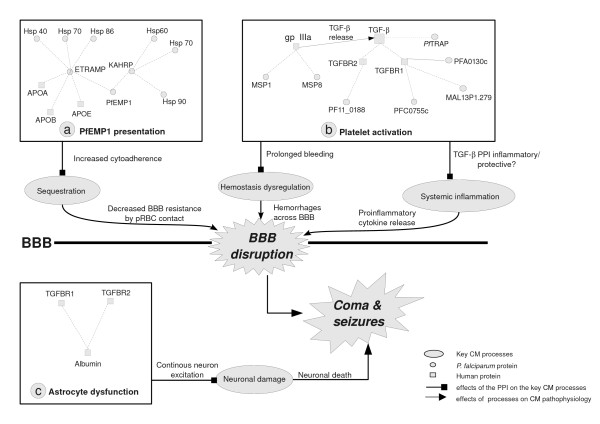
**Combined effects of specific PPI that could contribute to events and processes in CM pathology**. a) PfEMP1 presentation: Parasite Hsps - KAHRP/ETRAMP interactions during high temperature and the PPI between apolipoproteins and ETRAMP. b) Platelet activation: PPI between platelet membrane glycoprotein IIIa (gpIIIa) and merozoite surface proteins MSP1 and MSP8 resulting in TGF-β release. TGF-β released by activated platelets is activated by parasite protein PfTRAP. PPI between parasite proteins and TGF-β-TGF-β receptors TGFBR1, TGFBR2 might interfere in the anti-inflammatory role of TGF-β c) Astrocyte dysfunction: PPI between albumin and TGF-β receptors.

ETRAMP5 and the parasite Hsp protein PfHsp40 (PF14_0700) are also seen to interact. PfHsp40 has a direct interaction with PfHsp70 (PF08_0054) and an indirect interaction with PfHsp86 (PF07_0029). Other parasite Hsp proteins such as the PfHsp60 precursor, PfHsp70 and PfHsp90 also interact with various host proteins. The localization of ETRAMP5 to the TVN-pRBC membrane junction occurs via the chaperone activity of PfHsp70 and PfHsp86 and the co-chaperone PfHsp40 [26]. Another multi-chaperone complex consisting of PfHsp60 precursor, PfHsp70 and PfHsp90 traffics the knob-associated-histidine-rich protein (KAHRP) to the RBC membrane via the TVN [23,27]. In addition to ETRAMP5, KAHRP is also critical for efficient PfEMP1 presentation on the pRBC membrane [22,23,27]. Increased trafficking of PfEMP1 to the pRBC membrane leading to increased cytoadherence occurs during high temperature [28]. High temperature is also known to increase PfHsp expression and chaperone activity [28,29]. Thus, high temperature causes an increase in the expression and chaperone activity of PfHsps causing increased trafficking of KAHRP and ETRAMP5, leading to increased PfEMP1 presentation on the pRBC membrane. Figure 2a shows PPI associated with PfEMP1 presentation and sequestration.

#### Platelet activation

Glycoprotein integrin gpIIIa is seen to interact with the merozoite surface protein MSP-1. MSP-1 is initially expressed as a protein precursor, which undergoes primary proteolytic processing within the pRBC during late trophozoite and schizont stages resulting in four fragments. All the remaining fragments, except for the p19 Glycosylphosphatidylinositol (GPI) anchor present on the pRBC, are shed during schizogony to form a complex in association with MSP-6 and MSP-7 [30]. Platelets are known to be activated via membrane glycoprotein integrins such as gpIIb-IIIa [30,31]. It is known that parasite-derived products released at schizogony can act as triggers for platelet activation via platelet membrane glycoproteins followed by TGF-β release [31,32]. It can be hypothesized that the MSP-1 complex interacts with gpIIIa *in vivo *resulting in platelet activation. On the other hand, platelets can also get activated on contact with pRBCs [31]. It is possible that the MSP-1 GPI anchor on the pRBC surface interacts with gpIIIa during platelet-pRBC contact resulting in platelet activation. Thus, there are two possible *in vivo *scenarios for the MSP-1-gpIIIa interaction to occur.

A set of interactions between TGF-β and parasite proteins as well as those between TGF-β receptors and parasite proteins are observed. Regulation by parasite factors is through direct interactions involving TGF-β [33] or through interactions with platelets resulting in TGF-β release [32]. Experimental evidence suggests that PfTRAP (PF13_0201) activates latent TGF-β [33]. This activation is harmful during the early stages of the infection since TGF-β down-regulates the inflammatory cytokines resulting in reduced parasite clearance. However, in the later stages of the disease, TGF-β activation may be protective through the down-regulation of the systemic inflammation. Hence, the role of TGF-β could depend on the time of its activation by parasite proteins, with activation in early stages resulting in increased parasite clearance time. TGF-β released by activated platelets transduces signals by binding to TGF-β receptor type I (TGFBR1) and TGF-β receptor type II (TGFBR2) [34]. TGF-β receptor mediated signaling is crucial in endothelial apoptosis [35]. It is also known that the antagonistic binding of inhibitory proteins on TGF-β receptors interferes with TGF-β signaling and causes changes in the normal functioning of TGF-β [36,37]. Though the exact function of the parasite proteins involved in the PPI is currently unknown, it is possible that parasite proteins might interfere in TGF-β receptor mediated signaling. Figure 2b shows PPI associated with platelet activation and their probable linkage to haemostasis dysfunction/systemic inflammation.

#### Astrocyte dysfunction

An interaction between human serum albumin (HSA) and TGF-β receptors is also present in the landscape. The binding of HSA to astrocyte TGFBR1 and TGFBR2 following BBB disruption is associated with seizures in several neurological diseases [38,39]. HSA mediated astrocyte activation has been studied based on the up-regulation of glial fibrillary acidic protein (GFAP) [40], an intermediate filament protein that maintains mechanical strength of the astrocytes. Up-regulation of GFAP affects potassium and glutamate regulation by astrocytes [38]. Increased potassium and glutamate concentration in the vicinity of neurons causes neuronal death, leading to seizures caused by a reorganization of neuronal networks in the brain [40]. Figure 2c links this interaction to astrocyte dysfunction and neuronal damage.

## Discussion

This work aims at integrating host-parasite, host-host and parasite-parasite PPI from multitude of sources and using them to study the pathogenesis of CM. Despite a large corpus of literature on *P. falciparum *and human PPI from both experimental and theoretical sources, the *in vivo *significance of most of these PPI is not known. Since each PPI dataset has been derived using different experimental and theoretical methods, the interactions were initially treated as de-contextualized pairs of protein associations. GO annotation filters were then applied to the PPI data, which resulted in a set of host-parasite PPI that are most likely to influence CM pathogenesis. This filtering is dependent on the currently available GO annotations for the various parasite and host proteins, and as a result important protein interaction pairs could also get filtered out. The resulting PPI were then mapped to the key events PfEMP1 presentation, platelet activation and astrocyte damage resulting in a smaller, focused PPI set. Several established PPI not directly involved in these three key events get excluded. For instance, the interaction between PfEMP1 and CD36, which though responsible for increased cytoadherence, is filtered out as this event occurs only after PfEMP1 presentation.

The sequestration of pRBCs to the EC through the surface adhesion receptors [6] is crucial to CM, since this directly affects BBB structure and function. This process is known to occur mainly through interactions between the *P. falciparum *protein PfEMP1 and human proteins present on endothelial cells. PfEMP1 presentation is thus a key event in the sequestration process. Though KAHRP and ETRAMP are crucial in PfEMP1 presentation, the effect of temperature on the trafficking of these proteins by PfHsps is not fully understood. This work links interactions involving a set of PfHsps that might play a crucial role in the trafficking of ETRAMP and KAHRP to increased PfEMP1 presentation on the pRBCs. In CM, the effect of temperature in the trafficking of PfEMP1 to pRBC surface has been well studied [28]. Although antipyretics have been associated with reduced parasite clearance [41], the use of antipyretics in CM-afflicted patients is largely seen as protective [28]. This is attributed to decreased cytoadherence at lower body temperature, which assists anti-malarial drugs in clearing ring-stage parasites before they mature and cytoadhere. Further experimental work is necessary to study if this is due to the effect of antipyretics on the trafficking of ETRAMP and KAHRP resulting in decreased PfEMP1 presentation.

Activated platelets act as bridges between pRBCs and endothelial cells, allowing the binding of pRBC to the endothelium devoid of cytoadherence receptors [31]. The MSP-1-gpIIIa interaction might play a crucial role in platelet activation, either via complex formation with MSP-6/MSP-7 or through contact with pRBCs, thus influencing systemic inflammation in CM. Antibody mediated blocking of the activity of gpIIb-IIIa *in vitro *has revealed decreased platelet activation [31]. Other studies also show lowered antibody response to MSP-1/MSP-6/MSP-7 in CM patients when compared with non-affected patients [30]. Lowered antibody response to GPI anchor has also been reported in CM non-survivors when compared to the CM survivors [42]. Hence, the MSP-1-gpIIIa interaction could indicate a novel mechanism of platelet activation by MSP-1. Platelet activation is also associated with tissue factor (TF) expression on the platelet surface [43]. TF expression leads to amplification of the coagulation cascade resulting in the consumption of coagulation factors [6]. Platelet activation via MSP-1 and gpIIIa might thus also play a role in TF expression on the platelet surface causing amplification of the coagulation cascade, leading to haemostasis dysfunction.

Haemostasis dysfunction during CM culminates in increased endothelial hemorrhage resulting in leakage of plasma proteins, proinflammatory cytokines and parasite factors across the BBB. This influx of foreign substances activates the microglial cells, resident macrophages of the brain and spinal cord. Upon activation, they release proinflammatory cytokines which damage astrocytes and glial cells that are crucial for BBB maintenance [44]. In addition to the damage caused by this cytotoxic environment, it was hypothesized that the interaction between HSA and the TGF-β receptors TGFBR1 and TGFBR2 could result in astrocyte dysfunction, followed by seizures and neuronal death.

When mapping of the interactome, an interesting observation was the dual behaviour of TGF-β. On the one hand, it has a protective effect on the host during the pathogenesis of CM due to its anti-inflammatory property [34]. During the release of TGF-β mediated by parasite-derived products such as PfTRAP, TGF-β down-regulates the proinflammatory cytokine TNF and up-regulates the anti-inflammatory cytokine IL-10 [34]. On the other hand, activated platelets locally release TGF-β that synergizes with TNF in creating a proinflammatory condition leading to BBB disruption [33]. Increased activation of TGF-β during early stages of the disease leads to reduced parasite clearance time; hence it was hypothesized that parasite proteins might regulate TGF-β activation thus increasing parasite survival in the host.

## Conclusion

This work integrates disparate experimental and predicted host-parasite, host-host and parasite-parasite PPI into a combined interactome, filters this based on relevance to CM and positions the PPI around key events and processes of the disease. It points to the potential significance of apolipoproteins and Hsps on efficient PfEMP1 presentation, role of MSP-1 in platelet activation, the role of albumin in astrocyte dysfunction and the effect of parasite proteins in TGF-β regulation. The linking of these PPI to the molecular events associated with CM pathogenesis provides a basis for further experiments to determine the molecular basis of this fatal disease.

## Competing interests

The authors declare that they have no competing interests.

## Authors' contributions

MKK carried out the study and drafted the manuscript. TJ and GB participated in the design of the study, drafting of the manuscript and critically revising it. AR conceived the study, and participated in its design and coordination, as well as the drafting of the manuscript. All authors read and approved the final manuscript.
